# Early initiation of home-based sensori-motor training improves muscle strength, activation and size in patients after knee replacement: a secondary analysis of a controlled clinical trial

**DOI:** 10.1186/s12891-019-2575-3

**Published:** 2019-05-17

**Authors:** Maria Moutzouri, Fiona Coutts, John Gliatis, Evdokia Billis, Elias Tsepis, Nigel Gleeson

**Affiliations:** 1 Department of Physiotherapy, Technological Educational Institute (TEI) of Western Greece, Aigion, Greece; 2 School of Health Sciences, Queen Margaret University, Edinburgh, UK; 3 Orthopedic Surgery Department, University Hospital of Patras, Patras, Greece

**Keywords:** Exercise, Sensori-motor training, Neuromuscular capacity, Arthroplasty, Knee replacement

## Abstract

**Background:**

There is accumulating evidence for the advantages of rehabilitation involving sensori-motor training (SMT) following total knee replacement (TKR). However, the best way in which to deliver SMT remains elusive because of potential interference effects amongst concurrent exercise stimuli for optimal neuromuscular and morphological adaptations. The aim of this study was to use additional outcomes (i.e. muscle strength, activation and size) from a published parent study to compare the effects of early-initiated home-based rehabilitative SMT with functional exercise training (usual care) in patients undergoing TKR.

**Methods:**

A controlled clinical trial was conducted at the Orthopedic University Hospital of Rion, Greece involving allocation concealment to patients. Fifty-two patients electing to undergo TKR were randomised to either early-initiated SMT [experimental] or functional exercise training [control] in a home-based environment. Groups were prescribed equivalent duration of exercise during 12-weeks, 3–5 sessions of ~ 40 min per week of home-based programmes. Muscle strength and activation (peak force [PF]; peak amplitude [Peak Amp.] and root mean square of integrated electromyography [RMS iEMG]), muscular size (including rectus femoris muscle cross-sectional area [CSA_RF_]), and knee ROM were assessed on three separate occasions (pre-surgery [0 weeks]; 8 weeks post-surgery; 14 weeks post-surgery).

**Results:**

Patients undertaking SMT rehabilitation showed significantly greater improvements over the 14 weeks compared to control in outcomes including quadriceps PF (25.1 ± 18.5 N vs 12.4 ± 20.8 N); iPeak Amp. (188 ± 109.5% vs 25 ± 105.8%); CSA_RF_ (252.0 ± 101.0 mm^2^ vs 156.7 ± 76.2 mm^2^), respectively (*p* < 0.005); Knee ROM did not offer clinically relevant changes (*p*: ns) between groups over time. At 14 weeks post-surgery, the SMT group’s and control group’s performances differed by relative effect sizes (Cohen’s *d*) ranging between 0.64 and 1.06.

**Conclusion:**

A prescribed equivalent time spent in SMT compared to usual practice, delivered within a home-based environment, elicited superior restoration of muscle strength, activation and size in patients following TKR.

**Trial registration:**

ISRCTN12101643, December 2017 (retrospective registration).

**Electronic supplementary material:**

The online version of this article (10.1186/s12891-019-2575-3) contains supplementary material, which is available to authorized users.

## Background

Within Europe, more than 0.1% of national populations elect to undergo total knee replacements (TKR) annually [1–3] with 20–30% of patients dissatisfied with the outcome at the end of the pathway of care [4]. Patients’ underlying capacity to generate force and perform tasks does not fully recover until 12 months post-surgery [5, 6] and many patients may still present with pain, functional and balance limitations [7, 8]. Alternative conservative treatments may be better exploited to deliver better postoperative function and enhanced patient experiences and pathways of care that may counteract dissatisfaction after surgery [9]. Nevertheless, post-operative healthcare costs increase annually, with significant economic impact (£7000 [€7980] per patient over a 5-year follow-up period) [10].

The process of early active exercise in joint rehabilitation is significantly hindered by the patient’s inability to contract surrounding musculature (arthrogenic muscle inhibition (AMI), neural activation deficits linked to swelling, pain or structural damage), as is common after joint surgery [11, 12]. Functional rehabilitative training has conventionally incorporated muscle strengthening stimuli within functional weight-bearing exercises [13–16], but has not been capable of counteracting the post-surgery deficits in strength and disordered movement patterns [15–17].

The mode of exercise delivery during rehabilitation may be crucial to successful adaptation because eccentric-specific resistance exercise mitigates post-TKR deficits in strength [17–20], as does functionally-relevant sensori-motor training, with its emphasis on sensorial muscular coordination [21–27, 28]. A recently published parent study to the current one [22] showed that early initiation of novel SMT within a home-based environment counteracted contemporaneous deficits in sensori-motor function. It had focused on increased proprioceptive input in weight-bearing positions to improve motor responses dynamically and was adapted from antecedent work by Piva et al [25].

One of the unresolved issues in attempting to optimise post-TKR rehabilitative training is whether the reported additional gains in patients’ functional capacity associated with SMT are indeed due to mechanisms inherent within SMT or by means of increased physiological exposure to stimuli during greater volumes of exercise. This is because the majority [21, 25, 26] but not all studies [22], have relied upon unmatched comparisons of time spent in prescribed exercises to establish the superiority of SMT. Irrespective of the issues of quantifying the dose of SMT eliciting superior efficacy for developing functional capabilities post-TKR, an interaction of concurrent strength- and sensori-motor-focused stimuli appears to be better than conventional, strength-focused training at eliciting improvements in functionally-relevant capacities. While enhanced sensori-motor performance is considered to be positive because its linked causally to reduced prevalence of falls [22, 29, 30] and to the avoidance of other serious injuries [31, 32], the net outcome amongst physiologically-competitive and temporarily-adjacent stimuli during exercise, such as those for sensori-motor and strength, may be one of interference [33].

It is not yet known whether substantive gains in sensori-motor and functional capabilities [22] might be hindered by concomitant gains in strength. The aim of this study was to use partnering data associated with a previously published RCT investigation [22] to undertake a novel investigation of the patterning of gains amongst indices of strength performance and other neuromuscular and muscle size determinants of functional capability following early post-TKR initiation of home-based SMT. A secondary aim was to gain insight into the potential mechanisms by which strength performance is moderated during post-TKR rehabilitative care. We hypothesised that within a time-matched prescription of training, the SMT would provoke gains in direct (muscle strength) and indirect (integrated EMG) measures of neuromuscular performance and associated indices of muscle size (ultrasonic imaging) that would be inferior to those elicited by contemporary post-TKR rehabilitative practice with its greater emphasis on strength training.

## Methods

### Participants

Seventy consecutive patients (May 2012 – May 2014) undergoing primary standardised cemented unilateral TKR (single surgeon; 15-years’ experience of knee replacement; 50 knee replacements per annum) were invited to participate in the study. The inclusion criteria for participants were: a) Ambulatory at the time of surgery patients with OA (clinical and radiological findings of advanced osteoarthritis, 6–12 months length of wait for surgery) undergoing primary standardised cemented TKR by the same surgeon; b) Aged 65–80 years old. Patients were excluded from the study if they had: a) Infection, or complications after TKR; b) Neurological/neuromuscular conditions; c) Vestibular disorders that might affect balance; d) Other lower extremity orthopedic problems that limited function; e) Cardiovascular diseases, high blood pressure not controlled with medication and f) Unable to communicate or follow instructions or complete objective assessments.

A clinical trial was undertaken at a primary care university hospital in Greece (International Standard Randomised Control Trial Registration: ISRCTN12101643). All patients provided written informed consent, following a verbal and written explanation of the study procedures. The study had been ethically approved by two Institutional Committees (University Hospital of Patras, Greece and Queen Margaret University Edinburgh, UK [7052/4-7-2011]) and adhered to the Consort guidelines.

Ten blocks of 5 patients were randomly assigned to two groups (SMT [experimental]; Control [usual practice]) using a computer-generated number sequence overseen by an independent statistician. The confidential coded listing, maintained until after data analyses), assured allocation concealment from participants. This study involved a single-blind design as the principal investigator undertook assessment and training sessions. However, every effort was made to preclude bias (i.e. an undergraduate student recorded data during assessment sessions, and the principal investigator analysed data at the end of the rehabilitation period using the coded listing). A further 2 patients were included and assigned in the original block-allocation order, contributing to the study’s 52 patients.

### Time-matched rehabilitative procedure performed by both groups

All participants underwent a standardised post-surgery care-protocol, involving bedside physiotherapy and gait-retraining up to hospital-discharge (4–5 days after TKR) with hospital-based physiotherapists. After discharge, they were encouraged to continue the same exercise protocol and gait practice at home. A 12-week programme of self-managed, home-based exercises designed to enhance functional capabilities (modified from Piva et al [25]) was initiated at ~ 2 weeks after surgery (range 15–20 days). Additional file 1: Appendix 1 reproduced by an associated parent RCT study [22] details the delivery of SMT and control programmes. At the programme’s inception, an experienced physiotherapist (principal investigator) conducted an educational training session with patients in order to teach the key features and characteristics of safe delivery of the exercise programme that they would follow at home. Patients’ training programmes were further prescribed using a standardised illustrated guidebook of 14 exercises to regulate exercise-specific dosages. From week 3 to week 8, patients undertook 5 exercise sessions per week. Sessions increased progressively in duration from 35 to 45 min, incorporating progressively longer durations of walking from 10 to 20 min. Weeks 9 to 14 required patients to complete 45-min sessions of exercise, 3 times per week. The level of difficulty was progressed by adjusting exercise intensity to calibrate with weekly changes in each patient’s strength capability. Clinical oversight involved patients freely reporting effusion or discomfort and clarifying the delivery (accuracy, dose or safety) of the home-based exercises by telephone and by voluntary attendance ad libitum, for patients within both groups, within weekly scheduled clinical practical sessions. Patients’ compliance with the prescribed intensity, duration and frequency of exercise was verified by 7-day recall activity diaries. Experimental and control groups were prescribed identical procedures, number of exercises and total programme’ duration.

### Experimental group: sensori-motor training (SMT)

The experimental group undertook exercises that focused predominantly on enhancing sensori-motor functioning of patients. The SMT exercises included novel formulations of agility and perturbation training techniques [15, 16, 18, 19] which substituted for a proportion of training (50% – 7/14 exercises) within usual practice. Since the sensori-motor exercises were instructed to be delivered within a home-based environment, no specialised equipment was required. Exercise challenges and progression was achieved by using regular pillows to substitute for unstable surfaces, plastic cups for overcoming obstacles, and strategies such as bipedal to monopedal stance and eyes open to eyes closed in order to increase difficulty in maintaining or achieving balance.

### Control group

Usual care exercise sessions involved strengthening, stretching, and task-oriented functional exercises of the lower-extremity as described in other studies [15, 16, 34]. The content of the usual care programme was pragmatically adjusted to match the current trends of TKR rehabilitation [35].

### Outcome measures

The selected indices included measures of neuromuscular performance capability (muscle force and activation), muscle size and knee ROM. Randomly-ordered assessments of outcome data were collected by the principal investigator at pre-surgery, at 8 weeks post-surgery and at the study’s primary endpoint, 14 weeks post-surgery.

#### Neuromuscular performance capability

The knee extensors’ peak force (PF), measured in Newtons, was the study’s primary outcome and tested on an isokinetic dynamometer (Primus RS BTE, The Technology of Human Performance, USA). Muscle peak force (PF) was assessed during a maximum voluntary isometric contraction (MVIC) using a protocol adapted from Gleeson et al, [36]. The latter was recorded as the mean peak response from three intra-session muscle contractions. The reliability and reproducibility of assessing peak force has been verified [36–38].

Neuromuscular performance capability was assessed indirectly by surface EMG [38, 39] during MVICs (50 ms epoch, rectus femoris; Spike 2, version 5.16, Cambridge Electronics Design Ltd., UK). The EMG activity from the rectus femoris (RF) was recorded concomitantly with participants’ performance of static PF, using bipolar rectangular surface electrodes (self-adhesive, Ag/AgCl; 10 mm diameter; Unilect, UK). The root mean square (RMS) and peak amplitude, were used to describe the time-domain information of the EMG signal [40, 41], using commercially available software (Spike 2, version 5.16, Cambridge Electronics Design Ltd., UK). Normalisation of the EMG signal’s peak amplitude and RMS [42, 43] to the baseline MVIC (100%) facilitated inter-group comparisons over time. Reliability and validity of assessing EMG during MVICs has been verified by McKenzie et al. [42].

#### Knee ROM

Active range of flexion and extension movement (ROM) of the operated and non-operated knees was assessed by goniometry [43, 44] using the best of three attempts. The ICCs for flexion ROM has been found as 0.96, whilst for extension as 0.81 in a supine position [45, 46].

#### Muscle size

Muscle size alterations of the RF were examined throughout the study. Real time ultrasound image was captured at 7.1 MHz with a 55 mm linear probe (BK, mini focus, USA). Imaging was conducted in a seated position with the knee in 60° of flexion. Measurement of CSA_RF_ was undertaken using the method described by Bruin et al*,* [47]. Two images were captured with the muscle in maximum relaxation, and subsequently, another two images with the muscle during maximum voluntary isometric contraction (at the end of a 5 s contraction). Analysis of images was performed with ‘Image J’ software (https://imagej.nih.gov/ij/). Intra-rater reliability ICCs of 0.87 to 0.99 have been reported in studies measuring CSA_RF_ with a corresponding coefficient of variation (%) within the range from 3.5 to 8.9% [48, 49].

### Statistical analysis

The effects of the SMT were assessed *per protocol* for each outcome measure using separate factorial ANOVAs involving group (experimental; control) by leg (non-operated; operated) and by test occasion (pre-surgery; 8 weeks post-surgery; 14 weeks post-surgery) comparisons, with repeated measures on the latter two factors. Assumptions underpinning the use of ANOVA were assessed and corrections used Greenhouse-Geisser (GG), where appropriate. For outcomes that had focused on bilateral limb capabilities, group (experimental; control) by test occasion (pre-surgery; 8 weeks post-surgery; 14 weeks post-surgery) interactions were assessed, with repeated measures on the latter factor.

Effect size (ES; Cohen’s *d*) was calculated using pooled standard deviations [24]. A sample size of 30 participants per group had been computed a priori to discriminate moderate inter-group effects [14] at the study’s primary endpoint (14 weeks post-surgery) for its primary outcome (PF). Statistical significance was accepted at *p* < 0.05. Analyses used the Statistical Package for Social Sciences (SPSS; v. 16.0).

## Results

**Fig. 1:**
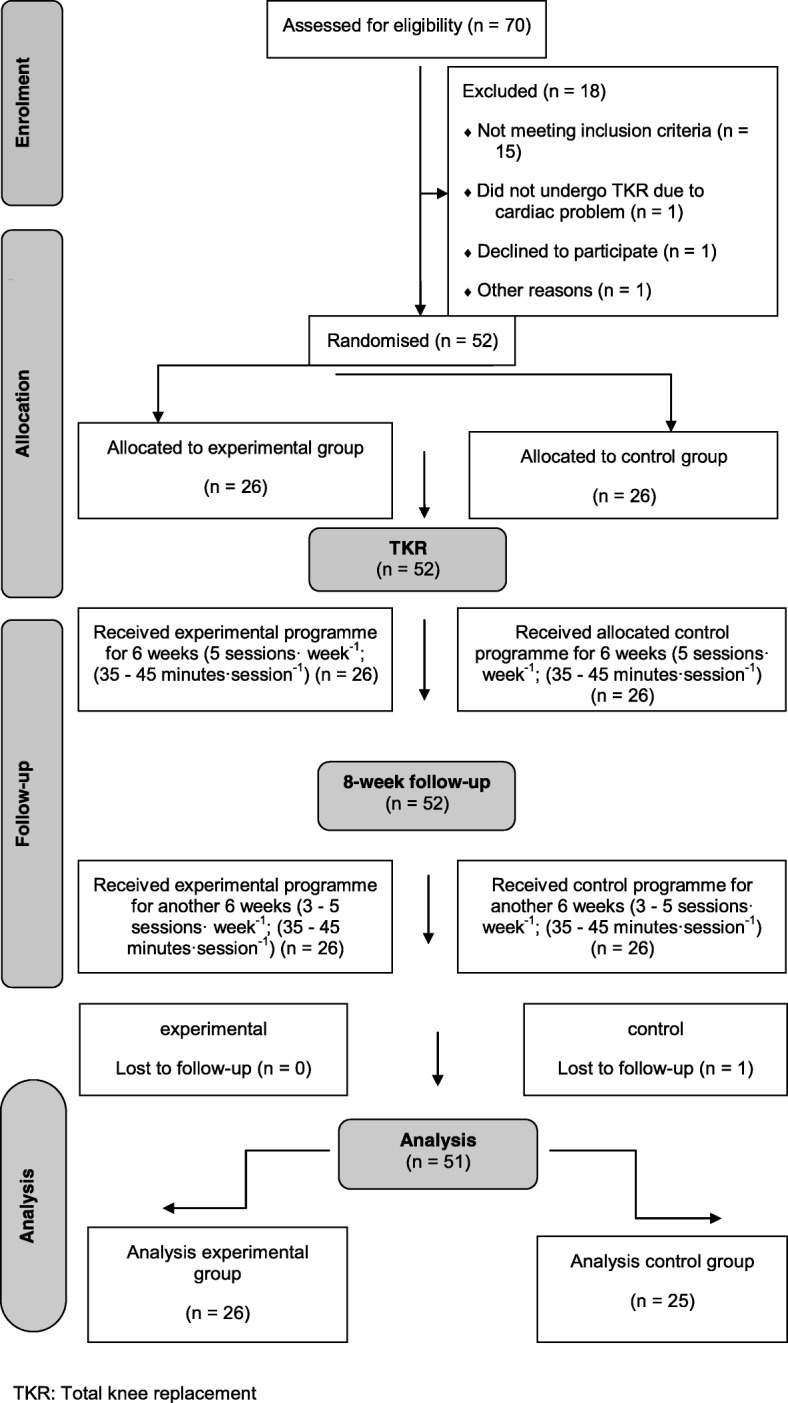
Patient CONSORT 1 flow of the study. (With permission to replicate adapted from Moutzouri et al, [22], Clinical Rehabilitation Journal)

Results for 51 of the 52 participants completing the study are reported (single patient exclusion for non-completion of the final assessment). A CONSORT flowchart and statistical equivalence of groups at baseline can be found in Fig. 1 and Table 1, respectively replicated by the parent study [22]. No adverse events were noted for any of the participants. Patients’ compliance to exercise showed an ~ 10% difference in favour of the SMT group.

**Table 1:** Pre-surgery (baseline) demographic characteristics, time on waiting list and measures of functional performance and pain

Variable	Group mean (SD)	
	Control (*n* = 25)	Experimental (*n* = 26)
Age (years)	72.3 (5.6)	71.3 (5.3)
Height (m)	1.64 (0.10)	1.66 (0.10)
Weight (kg)	82.1 (10.3)	82.5 (8.9)
Time to surgery (weeks)	17.2 (14.9)	15.3 (12.8)
TUG (s)	16.9 (3.8)	15.9 (3.6)
Pain (cm)	7.0 (1.1)	6.7 (1.2)

TUG: Timed Up and Go Test (with permission to reproduce, adapted from Moutzouri et al. [22], Clinical Rehabilitation Journal)

The SMT group yielded superior gains in muscle strength, activation and size compared to control (*p* < 0.001). Tables 2 and Table 3 show group mean scores for experimental and control groups, and for non-operated and operated legs, at baseline, 8 weeks post-surgery and 14 weeks post-surgery for outcomes of muscle strength and activation and for outcomes of knee ROM and muscle size, respectively.

**Table 2:** Group mean scores for the patients’ measures of muscle strength and activation across groups, for both limbs

Variable	Group	Pre-surgery	8 weeks	14 weeks	Change over time (12 weeks)				
Limb		Mean (SD)	Mean (SD)	Mean (SD)	Mean	F	*p* value	ES %	
iPeak Amp. (%) op.	control	100.0 (−)	108.8 (117.6)	126.3 (105.8)	26.3			–	26
non-op.		100.0 (−)	108.2 (77.2)	125.0 (63.6)	25.0			–	25
iPeak Amp. (%) op.	experimental	100.0 (−)	153.7 (160.8)	288.1 (109.4)	188.1	9.3	0.001**	–	188
non-op.		100.0 (−)	131.3 (88.4)	156.7 (92.3)	56.7			–	57
iRMS (%) op.	control	100. 0 (−)	143.7 (148.9)	181.2 (191.4)	81.2			–	81
non-op.		100.0 (−)	130.0 (92.3)	155.0 (169.2)	55.0			–	55
iRMS (%) op.	experimental	100.0 (−)	211.7 (141.0)	323.5 (157.1)	223.5	3.6	0.005*	–	223
non-op.		100.0 (−)	144.0 (160.0)	168.0 (136.1)	68.0			–	68
PF (N) op.	control	41.8 (17.8)	46.6 (18.6)	55.4 (23.5)	13.6			0.8	33
PF (N) non-op		57.2 (16.3)	60.0 (19.3)	69.1 (24.4)	11.9			0.7	21
PF (N) op.	experimental	39.8 (15.3)	49.3 (18.0)	67.5 (17.4)	27.7	1.3	0.47	1.8	28
PF (N) non-op		55.3 (20.6)	64.7 (24.4)	78.5 (20.9)	23.2			1.1	42

*p* value signifies the statistical significance of the main interaction effect between the groups; ES: relative effect size, computed as (group mean score at 14 weeks – group mean score at pre-surgery)/pooled SD; iP.Amp.: integrated EMG peak amplitude; iRMS: integrated Root Mean Square; PF: peak force; op.: operated limb; non-op.: non-operated limb; * *p* < 0.05; ** *p* < 0.001; ns: non-significant

**Table 3:** Group mean scores for the patients’ measures of muscle size and knee ROM across groups, for both limbs

Variable	Group	Pre-surgery	8 weeks	14 weeks	Change over time (12 weeks)				
Limb		Mean (SD)	Mean (SD)	Mean (SD)		F	*p* value	ES	%
ROM Flex (°) op.	control	106.9 (9.2)	101.3 (6.6)	103.7 (6.9)	−3.2	1.3	0.34	–	−2.9
non-op.		113.5 (10.5)	112.5 (9.0)	112.1 (8.5)	−1.4			–	− 1.4
ROM Flex (°) op.	experimental	105.1 (10.1)	104.4 (6.9)	107.3 (6.9)	2.2			0.02	2.2
non-op.		114.1 (9.8)	114.6 (9.0)	115.3(8.8)	1.2			0.01	1.0
ROM Ext (°) op.	control	−6.9 (5.2)	−4.2 (2.7)	−1.6 (0.9)	5.3	0.7	0.52	1.0	77
non-op.		−4.3 (4.1)	−3.1 (3.3)	−2.3 (1.5)	2.0			0.5	46
ROM Ext (°) op.	experimental	−6.0 (4.1)	−2.7 (2.1)	0.2 (1.1)	5.8			1.4	97
non-op.		−2.3 (2.4)	−1.5 (1.8)	−0.7 (1.5)	1.6			0.6	68
CSA_REL_ (mm^2^) op.	control	422.1 (72.0)	497.8 (69.4)	564.0 (91.1)	141.9			2.0	34
non-op.		460.9 (71.4)	537.3 (78.0)	612.1 (98.7)	151.2			2.1	33
CSA_REL_ (mm^2^) op.	experimental	450.2 (69.6)	557.9 (77.4)	708.6 (111.2)	258.4	19.6	0.001**	3.7	57
non-op.		487.4 (64.1)	578.5 (73.9)	663.9 (73.7)	176.5			2.7	36
CSA_CONTR_ (mm^2^) op.	control	338.1 (69.2)	433.0 (85.3)	494.8 (90.7)	156.7			2.3	46
non-op.		371.1 (66.9)	478.1 (80.4)	550.2 (94.9)	179.1			2.7	48
CSA_CONTR_ (mm^2^) op.	experimental	343.1 (67.8)	463.4 (86.9)	595.1 (128.4)	252.0	11.3	0.001**	3.7	73
non-op.		401.2 (65.3)	496.7 (84.6.)	576.3 (81.4)	175.1			2.7	44

*p* values signifies the statistical significance of the main interaction effect between the groups; ES: relative effect size, computed as (group mean score at 14 weeks – group mean score at pre-surgery)/pooled SD; op.: operated limb; non-op.: non-operated limb; ROM Ext: range of motion in extension; hypoextension = negative (−); hyperextension = positive; CSA_REL_: Cross-sectional area in rectus femoris in relaxation; CSA_CONTR_: Cross-sectional area of rectus femoris in contraction; ** *p* < 0.001

**Fig. 2:**
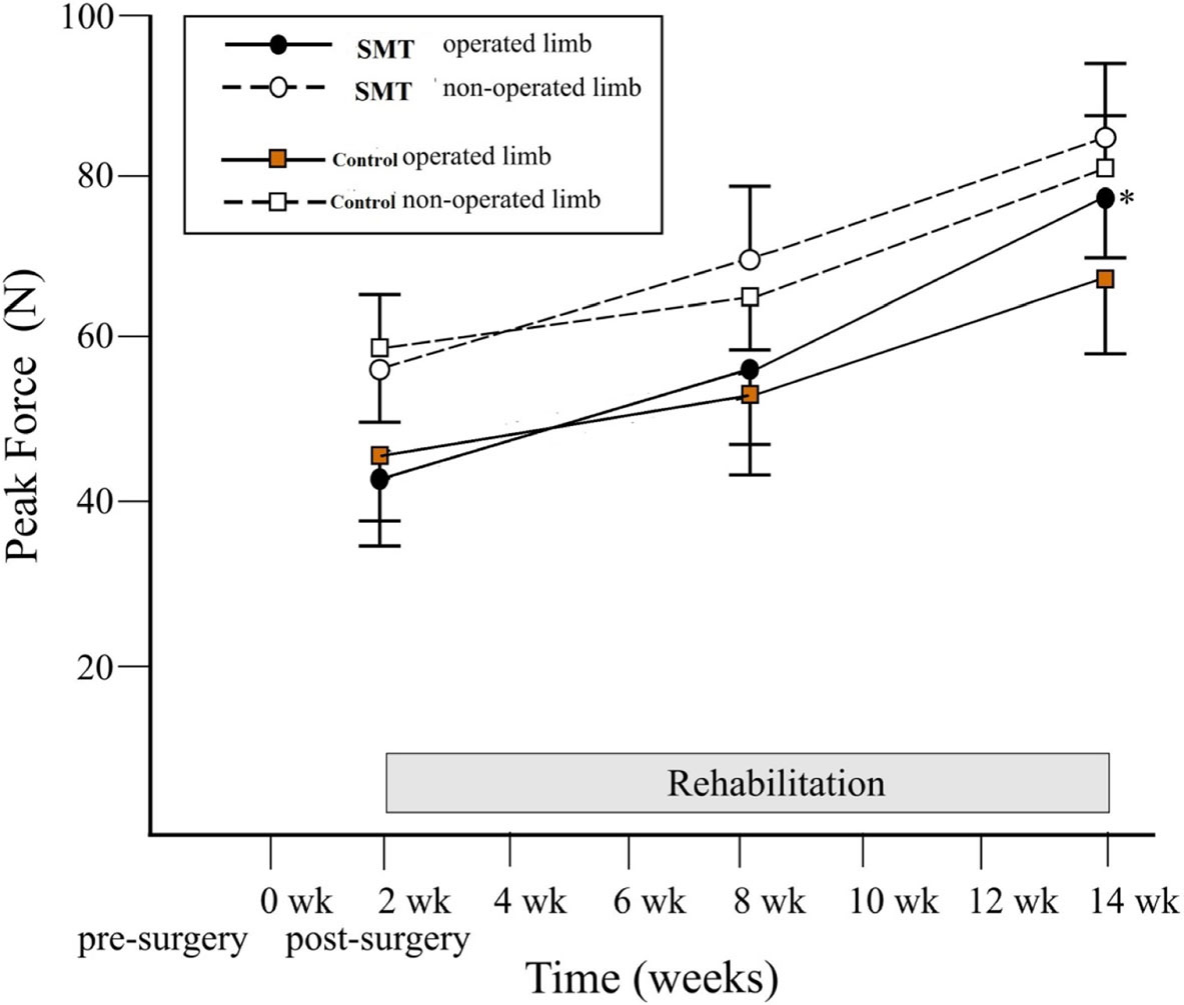
Muscle strength (peak force) scores of experimental and control groups for both the operated and non-operated legs from pre-surgery (0 weeks), 8 weeks and 14 weeks post-surgery, during TKR rehabilitation. TKR: total knee replacement; SMT: sensori-motor training group (experimental); Data represent mean ± 1SD; *p < 0.05, signifies interaction effect across groups over time

Sensori-motor training elicited superior gains in quadriceps muscle’ strength compared to control (PF: 25.1 ± 18.5 N vs 12.4 ± 20.8 N) as shown in Table 2 and Fig. 2. Interaction effect was significant across groups over time (F_(2, 98)_ = 7.15; *p* = 0.001), as improvements were similar for operated and non-operated legs.

Similarly, SMT was superior in eliciting gains in normalised peak amplitude (iPeak Amp.) (188 ± 109.5% vs 25 ± 105.8%) (F_(1.7,84.3)GG_ = 9.3; *p* < 0.001) and iRMS (223.5 ± 157.1% vs 81.0 ± 191.4%) (F_(1.9,93.6)GG_ = 3.6; *p* < 0.005) of the EMG signal, with the operated leg showing the most prominent improvements (Table 2).

Groups showed similar patterns of improvement over time on ROM during knee extension manoeuvres (F_(1.3, 66.8)GG_ = 0.65; ns) (Table 3). However, SMT elicited greater gains for knee flexion ROM (2.2 ± 6.9° vs − 3.2 ± 6.9°). Interaction effect was significant across groups over time (F_(1.2,61.2)GG_ = 5.6; *p <* 0.005), as improvements were similar for the operated and non-operated legs (Table 3).

Sensori-motor training elicited superior improvements in RF_CSA_ for both the relaxed and contracted states (60° of knee flexion), with a relatively greater improvement noted for the leg undergoing surgery (CSA_RF_ in relaxation: F_(1.6, 82.2)GG_ = 19.6, *p <* 0.001; CSA_RF_ in contraction_._: (CSA_RF_ (252.0 ± 101.0 mm^2^ vs 156.7 ± 76.2 mm^2^), F_(2, 98)_ = 11.3; *p <* 0.001) (Table 3).

## Discussion

The principal finding of this study was that SMT demonstrated superior gains in neuromuscular capacity compared to usual care, when the duration of prescribed training were matched. Thus, it would appear that the characteristics of novel sensori-motor training rather than its duration per se were important to enhancing post-TKR efficacy for improving strength and associated neurophysiological performance as measured by peak force and EMG amplitude and RMS. The pattern of enhanced gains in neuromuscular capacity associated with SMT within this study mimicked those noted previously for functional (TUG) and SM performance [22] (for example, ES for change in peak force [Cohen’s d] = 1.4; 52%: Table 2 vs. change in TUG performance [Cohen’s *d*] = 2.8; 49%, respectively). Importantly, these findings showed that the prior expectation of an unfavourable competitive interaction [33] between concurrent SM and strength stimuli within the SMT intervention had proved to be unfounded. In fact, it is physiologically plausible that given the prominent strength response to doses of focal SM training stimuli, the latter may have characteristics which are capable of physiologically potentiating the recovery of strength capacity. Only one other study to date corroborates SMT being capable of improving knee extensor muscle strength in patients with knee OA, albeit within a design involving non-matched duration of training [21].

The SMT’s efficacy may be driven by aspects of its content and dosage. It offers an emphasis on functional weight-bearing and balance/agility exercises in its content rather than standardised muscle stretching and strengthening exercises within usual care. The dosage of SMT mimics that of usual care (2–3 sessions/week) but the interaction of its exercises with the timing, intensity, duration and progression of training appears to potentiate an important cascade of gains in strength and functional mobility. As alluded to earlier, the time-matched prescription of exercise in this study potentially allows its efficacy in eliciting gains in strength in this study and concomitant functional capacities [22] to be attributed to the SMT’s characteristics and content.

Equivalent gains (~ 25%) in muscle strength for both the operated and non-operated legs elicited by SMT suggested that similar mechanisms were contributing to the process of training. Even with the potential intrusion of the physiological effects of AMI, there would appear to be no substantive impediment to the potential for patients to gain strength after TKR surgery. For example, while it’s expected that specific high intensity strength training initiated early following surgery (within two weeks) would have been capable of eliciting quadriceps muscle’ strength improvements of 7 and 30% at 12 and 26 weeks post-TKR in the operated limb, respectively [50], evidence from the current and previous [15, 27] studies show that SMT appears to offer some of the training stimuli necessary for potent gains in strength when delivered early after surgery, is perhaps more revelatory. Thus, the reasons for why many studies of usual care, with typically greater emphases on strength training, report being incapable of reversing strength loss for up to 6 months post-surgery [15, 16], remains somewhat elusive.

In the current study, the SMT group also showed concomitantly superior gains (~ 50%) in integrated EMG-derived outcomes (peak amplitude and RMS) that were more prominent for the operated leg. The latter suggested an expected physiological coupling of changes amongst indirect (muscle activation) and direct (muscle strength) measures of neuromuscular performance capability. It is plausible that SMT would have facilitated increased neural drive, numbers of active motor units, firing rates and other mechanisms for greater capacity in force generation. However, no significant relationships were noted amongst change scores for indices of peak force and the EMG-derived outcomes over the 14 weeks of the SMT programme (*p* > 0.05), which suggested that other factors had been important for determining gains in muscle strength.

The commonality amongst the extent of SMT-related changes for estimates of strength and neural drive extends further to encompass those for muscle architectural parameters. Using real-time ultrasound to assess muscle architecture’ parameters of TKR patients marked improvements were noted. These superior gains (~ 27%) were noted for CSA_RF_ during the contracted state at 60° of knee flexion for the experimental compared to the control group. The latter effect was more pronounced for the operated limb. This study’s CSA_RF_ changes were observed in both relaxed and contracted muscular states and endorse the potential importance of SMT to gaining muscle size. The patterns of SMT-related adaptations in performance for strength, activation and muscle size showed some concordance amongst the effect sizes. Nevertheless, a lack of correlation (*p* > 0.05) amongst the change scores for these outcomes suggested that primary determinants for the gains in the SMT group’s strength would not be clearly defined. A few studies have used CT scanning or MRI techniques (instead of ultrasound) to measure quadriceps’ muscle size along with muscle’ strength’ parameters [12, 17]. The study by Valtonen et al. [12] compared muscle strength and muscle size parameters between limbs and found a deficit of ~ 14% for the knee extensor muscle’ area of the operated side, 10 months after surgery. In the study by LaStayo et al. [17], increases of ~ 11% in quadriceps muscle’ size was observed following 12 weeks of eccentric training, compared to a control group, in which no changes were reported. The time-frame of follow-up (12 weeks) could be considered adequate for these considerable changes to have occurred, as the literature in the field suggests a minimum of 5 weeks of training before any morphological changes can be seen [51]. Therefore, findings from both the current study and from the literature suggest that enhanced motor-development strategies would need to be implemented for muscle size to show improved responses. The marked improvements in muscle size warrant further investigation in subsequent trials as to the author’s knowledge this was the first study reporting morphological changes with SMT.

A potential for assessor’s bias, and therefore potential overestimation of treatment effects, cannot be precluded due to the study’s single-blind design [52]. The current study has been limited to investigating the short-term effect of early-initiated SMT. Nevertheless, its novel findings, together with those from a parent study [22], suggests that compared to contemporary practice, the SMT has the potential to elicit superior recovery of functional performance in patients following TKR. Concomitant patterns of improvement and favourable interactions amongst sensori-motor, neuromuscular and muscle size capacities contribute to the mechanism by which the SMT achieved enhanced efficacy. Future research will establish the perseverance of the positive effects noted for SMT in this study and seek to identify optimised titrated dose-response characteristics. The latter will facilitate informed decision-making about SMT’s applicability within a wider range of environments. Future studies should also consider the calibration amongst substantive physical gains and any patient-perceived changes in physical capability, especially in self-managed care environments.

## Conclusion

In conclusion, a prescribed equivalent volume of time spent in SMT compared to usual practice, delivered within a home-based environment, elicited superior restoration of muscle strength, activation and size in patients following TKR. The gains in neuromuscular performance capability did not appear to be adversely influenced by patients responding predominantly to concurrent focal SM stimuli.

## Additional file

Additional file 1: **Appendix 1.** Comparison of exercise training programmes undertaken by the sensori-motor exercise training group (experimental) with the functional exercise training group (control). (DOCX 16 kb)

## Abbreviations

AMI: Arthogenic muscle inhibition
CSA: Cross sectional area
OA: Osteoarthritis
PF: Peak force
PROMs: Patient reported measures
QoL: Quality of life
RF: Rectus femoris
RMS: Root measn square
SMT: Sensori-motor training
TKR: Total knee replacement
TUG: Timed Up and Go
