# Supplementary material for: Early initiation of home-based sensori-motor training improves muscle strength, activation and size in patients after knee replacement: a secondary analysis of a controlled clinical trial
Source: BMC Musculoskelet Disord. 2019 May 17;20:231. doi: 10.1186/s12891-019-2575-3 (PMC6525469; doi:10.1186/s12891-019-2575-3)
Supplement: Supplementary file 1 — Appendix 1. Comparison of exercise training programmes undertaken by the sensori-motor exercise training group (experimental) with the functional exercise training group (control). (DOCX 16 kb) [file 12891_2019_2575_MOESM1_ESM.docx]

**Appendix 1**. Comparison of exercise training programmes undertaken by the sensori-motor exercise training group (experimental) with the functional exercise training group (control).

| **Exercise training programme** | **Sessional volume** | **Control** | **Experimental** |
| --- | --- | --- | --- |
| Ankle ROM | 10-20 reps | x |  |
| Knee ROM; Stretches 5-10 min. | 3-5 reps | x | x |
| Heel slide on wall | 10-20 reps | x |  |
| Straight leg raise | 3-5 sets of 10 reps | x |  |
| Quadriceps sets (short arc) | 3-5 sets of 10 reps | x | x |
| Quads strengthening with elastic band (sitting) | 3-5 sets of 10 reps | x | x |
| Quads strengthening with elastic band (standing ) | 3-5 sets of 10 reps | x |  |
| Hamstrings strengthening with elastic band (standing) | 3-5 sets of 10 reps | x |  |
| Abductors (side-lying) | 2-4 sets of 10 reps | x |  |
| Sit-to-stand | 10-20 reps | x | x |
| Wall slides | 10-20 reps | x | x |
| Calf raises | 10-20 reps | x |  |
| 20-30 min walking or stationary cycling | 5-20 min. | x | x |
| Climb on a platform of stairs | 10-30 steps | x | x |
| Marching (walk in place with large amplitude hip and knee flexion and upper limb movements) | 10-20 reps |  | x |
| Side stepping (step sideways, moving right to left and left to right) | 10-20 ft course length |  | x |
| Braiding activity (alternate steps front and back cross-over while moving laterally) | 10-20 ft course length |  | x |
| Square stepping | 10-20 ft course length |  | x |
| Walk over small obstacles | 10-20 ft course length |  | x |
| Balance on foam (place a foam or pillow and balance on two legs) | two- to single-leg stance |  | x |
| Tandem walking (bring one foot directly ahead of the other so that the heel of front foot touching toes of back foot) | 10-20 ft course length |  | x |

reps: repetitions. (With permission to reproduce, adapted from Moutzouri et al, [22], Clincal Rehabilitation Journal).
